# Intraspecific Responses of Seedlings of Three *Vachellia* Species to Simulated Browsing Reflect Adaptive Traits of Older Life Stages

**DOI:** 10.1002/ece3.71163

**Published:** 2025-03-18

**Authors:** Peter F. Scogings, Ntuthuko R. Mkhize

**Affiliations:** ^1^ Centre for Functional Biodiversity, School of Life Sciences University of KwaZulu‐Natal Scottsville South Africa; ^2^ School of Agricultural, Earth and Environmental Sciences University of KwaZulu‐Natal Scottsville South Africa

**Keywords:** *Acacia s.l.*, defense, herbivory, savanna, spinescence, tolerance

## Abstract

Intraspecific variation in adaptation to herbivory has been studied in juvenile (sapling) and adult (reproductive) stages of woody species in African savannas, but has not been studied at the early seedling stage. We hypothesized that, among *Vachellia* species commonly occurring in African savannas, spinescence increases and growth rate decreases after herbivory, but these responses would be expressed most strongly in populations with slower growing seedlings. Seedlings of *V. nilotica* , *V. tortilis*, and *V. karroo* were grown from seeds of different populations within the Southeastern Coastal Hinterland geomorphic province of South Africa. Seedlings were grown in a greenhouse and clipped at three intensities when they were 3 months old. Responses were determined for seedlings harvested 3 months later. Statistically significant (*p* < 0.05) interacting effects of clipping and population were rare. Clipping increased the spine mass fraction of *V. tortilis* seedlings from one population. Clipping reduced the relative height growth of *V. nilotica* and *V. tortilis* seedlings, while populations of *V. nilotica* and *V. karroo* differed in relative growth rate. We interpret weak vertical regrowth of *V. nilotica* and *V. tortilis* seedlings as reflecting adaptation to herbivory reported for saplings and adults in other studies. Conversely, we interpret strong height regrowth of *V. karroo* seedlings as reflecting adaptation to fire in association with herbivory or shading, as reported for older plants elsewhere. The study highlights the importance of studying plant traits relevant to herbivory in different populations and at different life stages to better understand adaptations to herbivory.

## Graphical abstract

Intraspecific variation in adaptation to herbivory has been studied in juvenile (sapling) and adult (reproductive) stages of woody species in African savannas, but has not been studied at the early seedling stage. Seedlings of *Vachellia nilotica* , *V. tortilis*, and V. karroo were grown from seeds of different populations, clipped at three intensities when 3 months old, and responses measured when 6 months old. We interpret weak vertical regrowth of *V. nilotica* and *V. tortilis* as reflecting adaptation to herbivory and interpret strong height regrowth of V. karroo as reflecting adaptation to fire in association with herbivory.

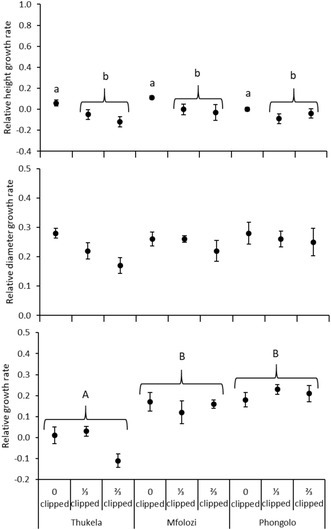

## 1 Introduction

Plants have many traits that reduce negative consequences on their fitness that may result from herbivory (Karban and Baldwin 1997). Some traits function as defenses that reduce the proximal degree of herbivory by deterring or repelling herbivores or by reducing herbivore abundance (Augner 1994; Karban and Baldwin 1997; Strauss and Agrawal 1999). Defensive traits include biochemical, anatomical, or architectural traits, such as phenolics, alkaloids, terpenoids, phytoliths, thorns, prickles, trichomes, small leaves, tough leaves, and dense branching (Zangerl and Bazzaz 1992; Berenbaum 1995; Freeland 1991; Harborne 1992; Kennedy and Barbour 1992; Lindroth 1989; Milton 1991; Myers and Bazely 1991; Pollard 1992; Vesey‐FitzGerald 1973; Pennings 1996). No defensive trait is ever completely effective against herbivores of plants expressing the trait due to “arms races” between plants and their herbivores (Ehrlich and Raven 1964). Thus, while under attack from herbivores or following an attack, some defensive traits may be induced (either enhanced or expressed de novo) to deter or repel subsequent herbivory or reduce herbivore abundance (Karban and Baldwin 1997).

Unlike defensive traits, which afford proximal protection against herbivores, tolerance traits reduce the ultimate negative consequences of herbivory through compensatory mechanisms that reflect a plant's ability to regrow and reproduce after herbivory (Mauricio et al. 1997; Painter 1951; Rosenthal and Kotanen 1994; Strauss and Agrawal 1999; Trumble et al. 1993; van der Meijden et al. 1988). Tolerance depends on physiological, anatomical, and morphological traits such as compensatory photosynthesis, availability of reserve carbohydrates for growth, rapid acquisition of new resources, adaptable resource allocation, availability and protection of buds, and adaptable phenology (Briske and Richards 1995; Rosenthal and Kotanen 1994; Strauss and Agrawal 1999; Tiffin 2000; Trumble et al. 1993). Plant genotypes with higher inherent growth rates are more able to tolerate herbivores (Stevens et al. 2007), but may be compromised by induced susceptibility to herbivores rather than induced defense (Herms and Mattson 1992; Lindroth and St Clair 2013). While defensive traits function in preventing herbivory, tolerance traits function in recovery after herbivory. As such, tolerance traits are unlikely to have the adverse effects on herbivore fitness that defense traits have and are thus unlikely to be affected by “arms races” (Koch et al. 2016; Rosenthal and Kotanen 1994; Stowe et al. 2000; Strauss and Agrawal 1999).

Investment of resources into defensive traits or tolerance traits varies according to life stage because of different selection pressures experienced at different stages and differences in other factors such as resource availability and allocation (Barton 2008; Boege et al. 2007). The seedling stage is generally considered most susceptible to herbivory because seedlings are more accessible but developmentally constrained (Barton 2008; Moles and Westoby 2004; Staver et al. 2009; Walker 1985). Tolerance is generally weaker in seedlings than in older plants (Barton 2016; Boege et al. 2007; Massad 2013; Rosenthal and Kotanen 1994), but defense is generally stronger, especially in herbivore‐rich ecosystems (Armani et al. 2019; Boege et al. 2007; Brooks and Owen‐Smith 1994; Bryant et al. 1983). Weaker tolerance in seedlings can result from insufficient time to build up reserves of carbohydrates and buds for regrowth, while stronger defense can result from multiple functions of secondary metabolites (Boege et al. 2007; Herms and Mattson 1992).

Variation in a plant species' adaptations to herbivory also exists among populations because of different selection pressures (Heywood 1991; Loveless and Hamrick 1984; Wang and Bradburd 2014). For example, populations of *Vachellia karroo* (Hayne) Banfi & Galasso express different architectures under different selection pressures because of a trade‐off between apical and lateral growth, known as Corner's Rule (Ackerly and Donoghue 1998; Archibald and Bond 2003). Populations adapted to high browser pressure allocate resources to enhanced activation of lateral buds after herbivory, leading to increased branching (Archibald and Bond 2003). The resulting ‘cage’ or ‘dome’ architecture is defensive because it restricts access to browseable material (Bilbrough and Richards 1991; Charles‐Dominique et al. 2017; Vesey‐FitzGerald 1973). Populations exposed to high fire frequency allocate resources to apical buds, enabling rapid height growth and resulting in “pole” architecture (Archibald and Bond 2003). Expression of pole architecture has additional benefits when the fire is associated with other pressures, such as browsing or shading (Allcock and Hik 2004; Archibald and Bond 2003; Higgins et al. 2000; Staver et al. 2009; Stevens et al. 2007; Trollope 1984; Vandenbussche et al. 2005; Wakeling et al. 2011). Such intraspecific variations in defense and tolerance must be considered when studying plant–herbivore interactions (Barton and Boege 2017; Gols et al. 2019).

Savannas are tropical and subtropical ecosystems that have distinctive rainy and dry seasons and are characterized by an herbaceous layer dominated by heliophilous C_4_ graminoids and a discontinuous, diverse woody layer (Archibald et al. 2020; Frost et al. 1986). The assemblage of the woody layer is strongly affected by seedling establishment, which is particularly constrained by herbivory within the rainy season in which germination happens (Archibald et al. 2021). Shading from herbaceous plants is another factor constraining woody seedling establishment (Chirara 2001; Smith and Shackleton 1988). In the subsequent dry season, seedling survival is constrained by different factors, especially water stress or fire, which require that resources acquired in the first growing season be allocated preferentially to rapid primary growth, that is, root or shoot elongation, before the dry season starts (Archibald et al. 2021; Swemmer and Ward 2020). Early herbivory, therefore, poses a convergence of potentially opposing priorities with high certainty of grave consequences (Archibald et al. 2021). While invertebrates and small mammals are important herbivores of seedlings, large mammal herbivores are particularly relevant in African savannas (Augustine et al. 2020; Goheen et al. 2004; Shaw et al. 2002).

*Vachellia* Wright & Arn. is a widely distributed genus in savannas of Africa, western and southern Asia, and Central and South America. Several *Vachellia* species are known woody encroachers of grasslands and savannas, such that some are declared weeds (Comben et al. 2021; Tedder et al. 2012). Within species, populations that predominantly express tolerance of browsing or fire, for example, rapid vertical growth at the seedling stage, could more successfully encroach into grasslands than populations that predominantly express defensive traits, for example, spinescence (Venter et al. 2018; Wakeling et al. 2011). Intraspecific variation in adaptation to herbivory is evident in juvenile and adult life stages (Archibald and Bond 2003) but has not been studied at the very early seedling stage. Therefore, we aimed to explore this gap. Our objectives were (i) to determine responses to simulated herbivory of seedlings of three *Vachellia* species commonly found in southern Africa and (ii) to determine intraspecific variations among seedlings from different populations. We hypothesized that spinescence increases and growth rate decreases after clipping, especially in populations expressing slower growth rates of seedlings (Boege et al. 2007; Stevens et al. 2007).

## 2 Materials and Methods

### 2.1 Study Area and Species

The Southeastern Coastal Hinterland geomorphic province of South Africa was selected for the study because the area presented an opportunity to explore variation in plant traits relevant to herbivory in populations of *Vachellia* species that may have been geographically isolated from each other for millions of years because of tectonic uplift events (Scogings and Mkhize 2023). The Southeastern Coastal Hinterland extends along ~1000 km of the ~150 km‐wide eastern seaboard of southern Africa (Knight and Grab 2015; Partridge et al. 2010). Tectonic uplift events that occurred ~20 million years ago (Ma) and ~5 Ma resulted in the Southeastern Coastal Hinterland becoming dissected by several drainage basins in which rivers are deeply incised in their middle and lower reaches (Knight and Grab 2015; Partridge and Maud 1987; Partridge et al. 2010). Major river valleys tend to be drier and warmer (500–900 mm mean annual precipitation, MAP; 17.8°C–20.8°C mean annual temperature, MAT) than the watersheds between basins (600–1000 mm MAP; 17.2°C–18.8°C MAT) (Rutherford, Mucina, Lötter, et al. 2006).

Grassland is the main vegetation of inland areas of the Southeastern Coastal Hinterland (> 80 km from the coast), while vegetation nearer the coast is mainly savanna comprising dense “thornveld” or “bushveld” in valleys and sparsely scattered trees in grasslands on watersheds (Rutherford, Mucina, Lötter, et al. 2006; Rutherford et al. 2006; https://archive.org/details/vegetationofsout19muci/page/n513/mode/2up accessed on 7 January 2022). *Vachellia* species occur in these savannas, but their diversity and abundance are much greater in the valleys than in watersheds (Rutherford, Mucina, Lötter, et al. 2006). Of the widespread species, *V. tortilis* (Forssk.) Galasso & Banfi is restricted to valleys, while *V. karroo* and *V. nilotica* (L.) P.J.H. Hurter & Mabb. occur across valleys and watersheds but at much greater abundances in valleys (Rutherford, Mucina, Lötter, et al. 2006). All three species are phenotypically diverse throughout their global distributions (El Ferchichi Ouarda et al. 2009; Taylor and Barker 2012; Wolde‐meskel and Sinclair 1998). Genetic diversity of *V. nilotica* and *V. tortilis* , which are distributed throughout Africa, western Asia, and southern Asia, is also high, and this is suggested by some studies to be in response to the geographic isolation of populations (Kyalangalilwa et al. 2013; Omondi et al. 2019; Taylor and Barker 2012). In contrast, *V. karroo* is only widely distributed in southern Africa and lacks genetic diversity despite high phenotypic diversity, possibly resulting from recent, rapid evolution that has not yet produced genetic differentiation (Taylor and Barker 2012; Tsvuura and Ward 2022).

### 2.2 Seed Collection

Seeds of each species were collected from multiple parent plants at least 100 m apart from each other within sites in the middle reaches of major rivers in the central section of the Southeastern Coastal Hinterland (table 1; figure 1 in Scogings and Mkhize 2023). Altitudes of the sites from which seeds were collected ranged from 305 to 895 m above sea level, while MAP was 632–894 mm and MAT was 17.6°C–21.6°C (Table 1). Geology and pedology across sites were similar because they were in valleys incised through Natal Group sandstones overlying older Namaqua‐Natal Metamorphic Province granites and gneisses (Partridge and Maud 1987).

**TABLE 1:** Coordinates, altitude, mean annual precipitation (MAP), and mean annual temperature (MAT) of the sites from which seeds of three *Vachellia* species were collected (from Scogings and Mkhize 2023).

Site	Species	Latitude (°south)	Longitude (°east)	Altitude (m)	MAP (mm)	MAT (°C)
Mkomazi	*V. karroo*	−29.90	30.07	895	803	17.6
Mgeni	*V. karroo*	−29.64	30.49	660	813	18.2
Thukela	*V. karroo* *V. nilotica* *V. tortilis*	−28.74	30.26	635	655	19.2
Mfolozi	*V. nilotica* *V. tortilis*	−28.35	31.40	440	894	19.6
Phongolo	*V. nilotica* *V. tortilis*	−27.35	31.78	305	632	21.6

Seeds of *V. nilotica* subsp. *kraussiana* (Benth.) Kyal. & Boatwr. and *V. tortilis* subsp. *heteracantha* (Benth.) Kyal. & Boatwr. were collected from the Thukela, Mfolozi, and Phongolo valleys, and seeds of *V. karroo* were collected from the Thukela, Mgeni, and Mkomazi valleys. The selection of these valleys permitted comparisons of seedling populations that differ in growth rates. In a companion study over 3 months at the same time and in the same environment, seedlings of *V. tortilis* and *V. nilotica* from Phongolo grew taller than those from Thukela, which was hypothesized as an adaptation to a stronger evolutionary history of browsing and fire at Phongolo compared to other valleys (Scogings and Mkhize 2023). In contrast, seedlings of *V. karroo* from Thukela, Mgeni, and Mkomazi did not differ in height, but those from Thukela had thicker stems than those from Mgeni. Seedlings of *V. nilotica* from Thukela (the site where all three species were collected) grew noticeably faster than seedlings of *V. tortilis* and *V. karroo* from Thukela (Scogings and Mkhize 2023).

### 2.3 Seedling Establishment

Between 15 and 20 seeds per site (population) per species were sterilized for 15 min in NaOCl, rinsed thoroughly with distilled H_2_O, and scarified using nail clippers. Agar‐agar powder (8 g) in 1000 mL H_2_O was mixed and then boiled for 10 min. The solution was allowed to solidify for 3 min in petri dishes before seeding the gel and sealing the dishes against contamination. Germination was allowed to happen at room temperature, and when seedlings were 5 days old, they were transplanted into pots (20 cm diameter) filled with river sand. Pots with seedlings of a species from a single site were randomly assigned to three intensities of simulated browsing (control, moderate, severe). All pots were then arranged in a completely randomized design within each species in a greenhouse, and seedlings were grown for 6 months, watering twice weekly. Humidity in the greenhouse was 50%–70%, while maximum daily temperatures were 23°C–35°C and minimum daily temperatures were 9°C–16°C.

### 2.4 Simulated Browsing

Three months after potting, half the seedlings in the clipping treatments were clipped with scissors to remove the upper one‐third of the stem, and half were clipped to remove the upper two‐thirds to represent moderate and severe browsing, respectively. Whether the decapitation of such young seedlings (~70 mm tall) is done by a single, instantaneous snip of scissors or a browsing mammal's teeth was assumed to be trivial because no complex decisions by a herbivore feeding on larger plants needed to be simulated. Seedlings grew for 3 months after treatment, and then, seedling height (mm) and basal stem diameter (mm) were measured on the 6‐month‐old plants. Seedlings were harvested, oven‐dried at 60°C for 48 h, and weighed (mg). Spines on each seedling were counted and weighed. Leaf mass fraction (LMF = leaf dry mass/total dry mass) was determined because of its relationship to relative growth rate (RGR, Hoffmann and Poorter 2002) of deciduous species in savannas, and it relates negatively to carbohydrate storage (Tomlinson et al. 2014). Spine mass fraction (SMF) was calculated as for LMF. Average height, diameter, and total dry mass at the time of treatment were obtained from seedlings that were germinated and potted at the same time as our seedlings and grown alongside them in a companion study (Scogings and Mkhize 2023). These were used to calculate relative height growth rate (RHR, mm mm^−1^ month^−1^), relative diameter growth rate (RDR, mm mm^−1^ month^−1^), and relative growth rate (RGR, mg mg^−1^ month^−1^) following Hoffmann and Poorter (2002) and Wakeling et al. (2011).

### 2.5 Data Analysis

Sample size at harvesting was five to six seedlings per treatment (depending on mortalities during the experiment). These mortalities were infrequent and occurred randomly across treatments, indicating they were unrelated to treatment effects. Sample sizes were restricted because greenhouse space was limited (between this study and its companion, there were > 250 seedlings to manage altogether). Spine mass fraction of *V. nilotica* and *V. tortilis* was normalized by square‐root transformation. LMF, RHR, RDR, and RGR were normally distributed for all species. Within each species, the effects of site, clipping, and their interaction were tested using two‐way MANOVA to limit the experiment‐wise error rate of multiple ANOVAs. Type III sums of squares allowed for unequal sample sizes. Means were separated using Tukey's HSD test. Significance was declared when *p* < 0.05.

## 3 Results

**FIGURE 1:**
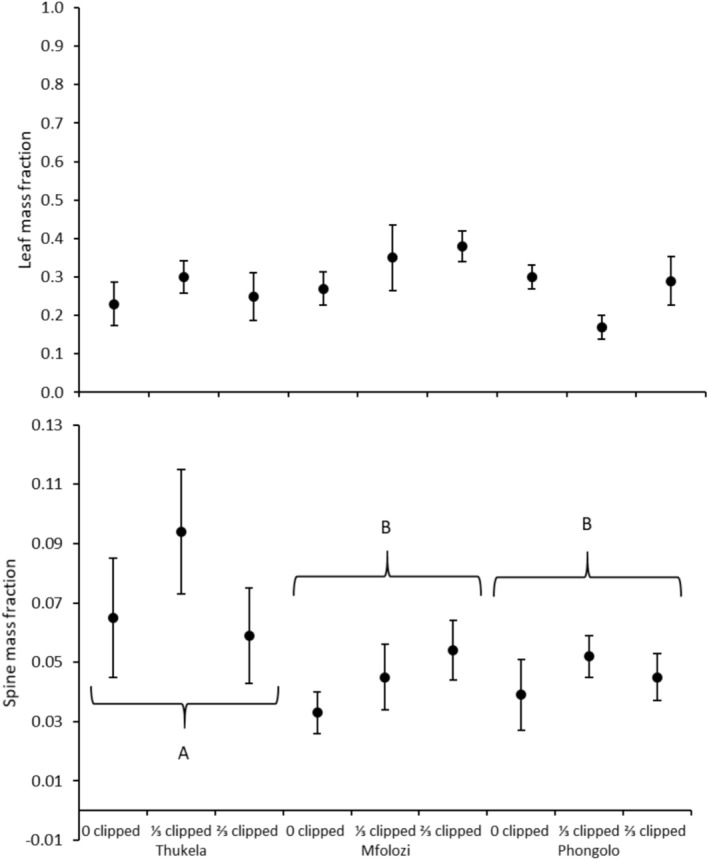
Mean leaf mass and spine mass fraction of *Vachellia nilotica* seedlings grown from seeds collected in three major river systems in the Southeastern Coastal Hinterland geomorphic province of South Africa. Error bars are standard errors of means. Different uppercase letters indicate significant differences between sites according to Tukey's HSD test (*p* < 0.05).

**FIGURE 2:**
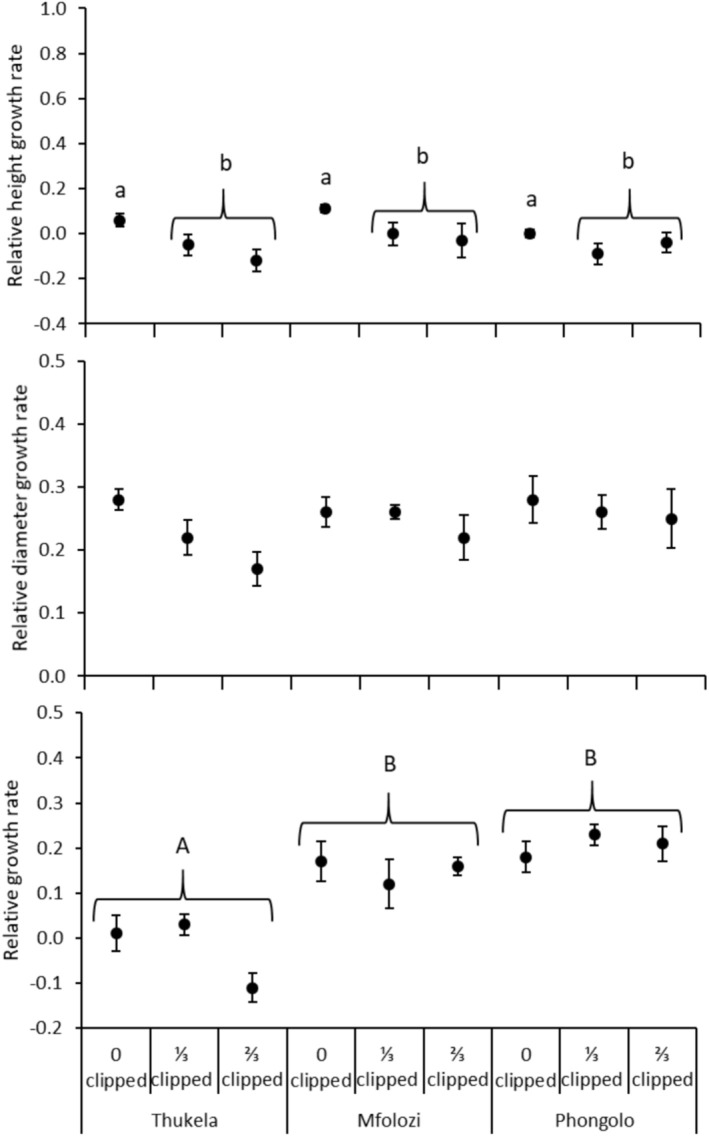
Mean relative height growth rate (mm mm^−1^ month^−1^), relative diameter growth rate (mm mm^−1^ month^−1^), and relative growth rate (mg mg^−1^ month^−1^) of *Vachellia nilotica* seedlings grown from seeds collected in three major river systems in the Southeastern Coastal Hinterland geomorphic province of South Africa. Error bars are standard errors of means. Lowercase letters within a site indicate significant differences between clipped versus control plants and uppercase letters indicate significant differences between sites according to Tukey's HSD test (*p* < 0.05).

No significant interacting effects of clipping and site on response variables were detected in *V. nilotica* seedlings (*F*_24,140.75_ = 1.248; *p* = 0.212; Wilk's Lambda = 0.510). However, main effects were significant (Site: *F*_12,80_ = 5.850; *p* < 0.001; Wilk's Lambda = 0.284 | Clipping: *F*_12,80_ = 2.491; *p* = 0.008; Wilk's Lambda = 0.530). Seedlings grown from seeds originating from Thukela had higher SMF than those originating from Phongolo (Table 2; Figure 1), while seedlings originating from Mfolozi and Phongolo grew faster than those originating from Thukela (Figure 2). Both clipping levels significantly reduced RHR compared to unclipped plants (Table 2; Figure 2).

**TABLE 2:** Analysis of variance of number of spines, leaf mass fraction (LMF), square root spine mass fraction (SMF), relative height growth rate (RHR, mm mm^−1^ month^−1^), relative diameter growth rate (RDR, mm mm^−1^ month^−1^), and relative growth rate (RGR, mg mg^−1^ month^−1^) of *V. nilotica* seedlings (*n* = 54) grown in pots for 3 months following three clipping treatments applied at 3 months of age.

Dependent	Independent	Type III SS	df	Mean squares	*F*‐ratio	*p*
LMF	Clipping	0.014	2	0.007	0.399	0.674
	Site	0.073	2	0.037	2.157	0.127
	Clipping*Site	0.104	4	0.026	1.525	0.211
	Error	0.764	45	0.017		
SMF	Clipping	0.016	2	0.008	1.501	0.234
	Site	0.042	2	0.021	3.960	0.026
	Clipping*Site	0.013	4	0.003	0.626	0.646
	Error	0.237	45	0.005		
RHR	Clipping	0.152	2	0.076	6.265	0.004
	Site	0.055	2	0.027	2.253	0.117
	Clipping*Site	0.039	4	0.010	0.797	0.534
	Error	0.544	45	0.012		
RDR	Clipping	0.032	2	0.016	3.031	0.058
	Site	0.014	2	0.007	1.334	0.274
	Clipping*Site	0.012	4	0.003	0.582	0.677
	Error	0.234	45	0.005		
RGR	Clipping	0.018	2	0.009	1.129	0.332
	Site	0.515	2	0.257	33.087	< 0.001
	Clipping*Site	0.071	4	0.018	2.269	0.076
	Error	0.350	45	0.008		

**FIGURE 3:**
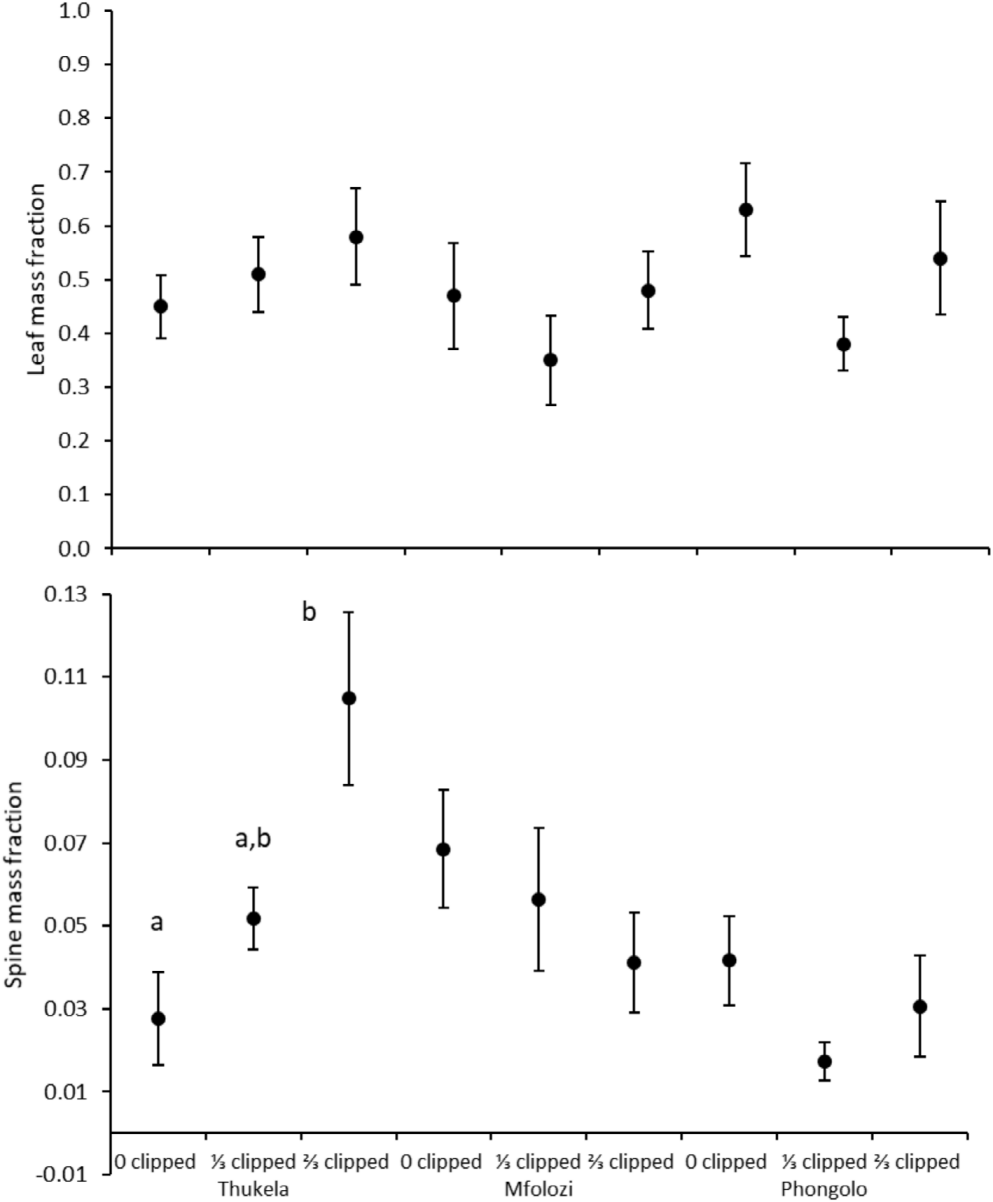
Mean leaf mass and spine mass fraction of *Vachellia tortilis* seedlings grown from seeds collected in three major river systems in the Southeastern Coastal Hinterland geomorphic province of South Africa. Error bars are standard errors of means. Lowercase letters within a site indicate significant differences between clipped versus control plants (none indicate no difference) according to Tukey's HSD test (*p* < 0.05).

**FIGURE 4:**
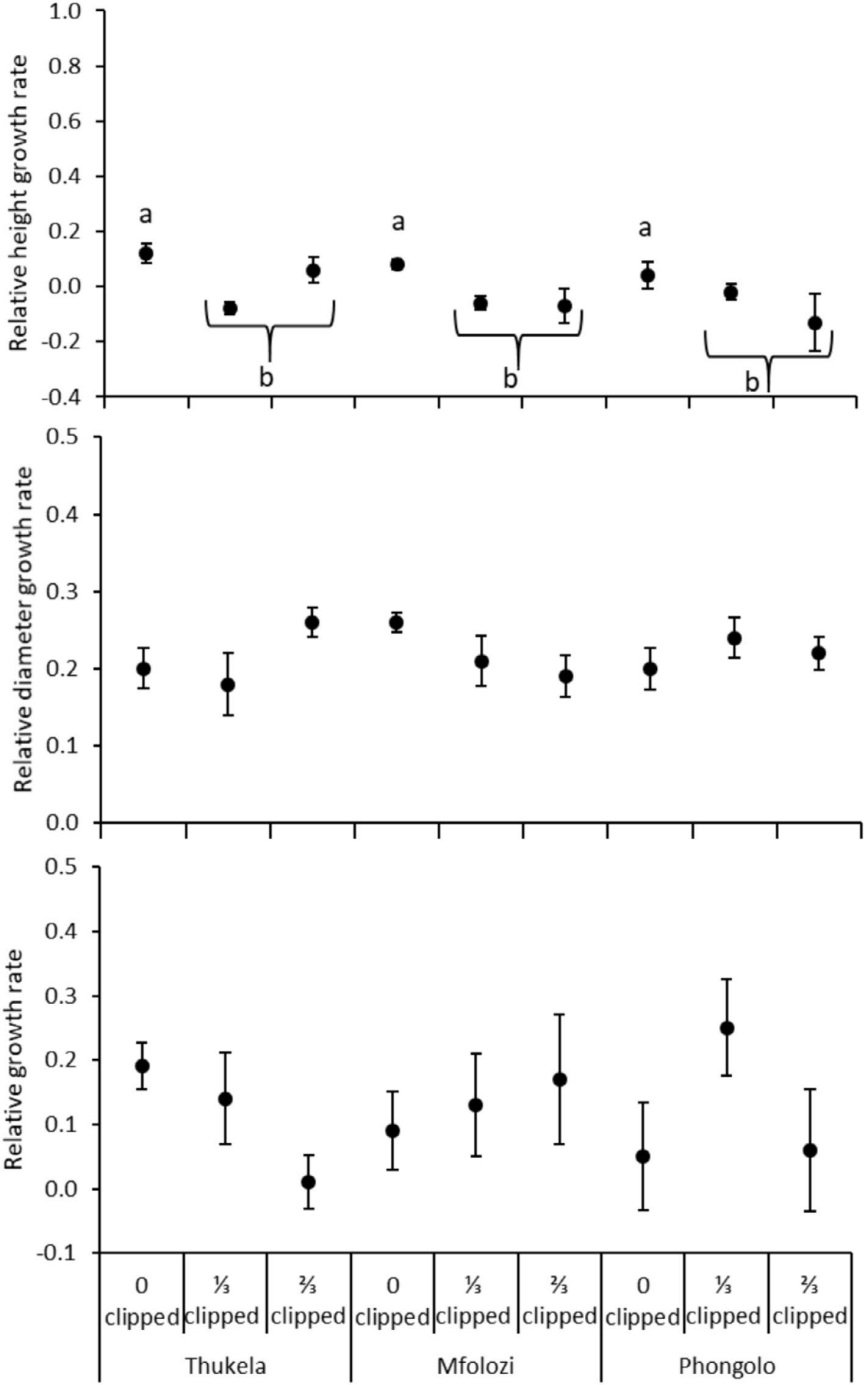
Mean relative height growth rate (mm mm^−1^ month^−1^), relative diameter growth rate (mm mm^−1^ month^−1^), and relative growth rate (mg mg^−1^ month^−1^) of *Vachellia tortilis* seedlings grown from seeds collected in three major river systems in the Southeastern Coastal Hinterland geomorphic province of South Africa. Error bars are standard errors of means. Lowercase letters within a site indicate significant differences between clipped versus control plants according to Tukey's HSD test (*p* < 0.05).

Significant interaction between clipping and site on the response variables was detected for *V. tortilis* seedlings (*F*_20,123.67_ = 1.871; *p* = 0.020; Wilk's Lambda = 0.416). Eigenvalues indicated that SMF contributed most to the variation in the combined variables. Compared to unclipped plants, the most severe clipping level significantly increased the SMF of seedlings originating from Thukela but had no significant effect on SMF of seedlings originating from other sites (Table 3; Figure 3). Compared to unclipped plants, both clipping levels significantly reduced RHR (Table 3; Figure 4). Site had no independent effects on any response variables (Table 3; Figures 3 and 4).

**TABLE 3:** Analysis of variance of leaf mass fraction (LMF), square root spine mass fraction (SMF), relative height growth rate (RHR, mm mm^−1^ month^−1^), relative diameter growth rate (RDR, mm mm^−1^ month^−1^), and relative growth rate (RGR, mg mg^−1^ month^−1^) of *V. tortilis* seedlings (*n* = 50) grown in pots for 3 months following three clipping treatments applied at 3 months of age.

Dependent	Independent	Type III SS	df	Mean squares	*F*‐ratio	*p*
LMF	Clipping	0.169	2	0.084	1.926	0.810
	Site	0.079	2	0.039	1.074	0.351
	Clipping*Site	0.135	4	0.034	0.922	0.460
	Error	1.504	41	0.037		
SMF	Clipping	0.008	2	0.004	0.816	0.449
	Site	0.053	2	0.027	5.483	0.008
	Clipping*Site	0.135	4	0.021	4.416	0.005
	Error	0.199	41	0.005		
RHR	Clipping	0.198	2	0.099	7.684	0.001
	Site	0.043	2	0.021	1.670	0.201
	Clipping*Site	0.086	4	0.021	1.668	0.176
	Error	0.527	41	0.013		
RDR	Clipping	0.003	2	0.001	0.322	0.727
	Site	0.001	2	0.000	0.093	0.911
	Clipping*Site	0.033	4	0.008	2.101	0.098
	Error	0.161	41	0.004		
RGR	Clipping	0.076	2	0.038	1.210	0.309
	Site	0.002	2	0.001	0.036	0.965
	Clipping*Site	0.175	4	0.044	1.385	0.256
	Error	1.293	41	0.032		

**FIGURE 5:**
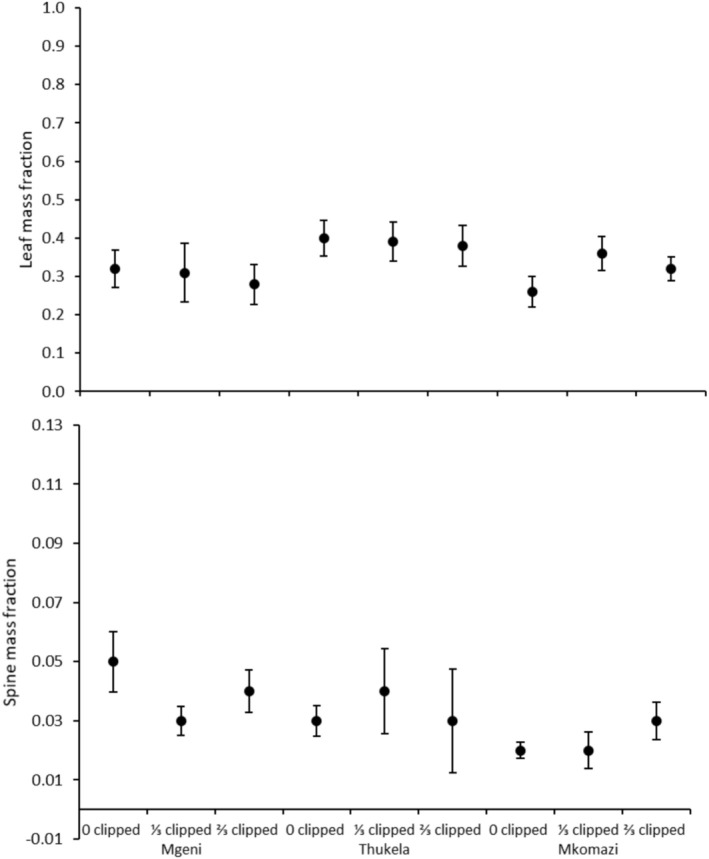
Mean leaf mass and spine mass fraction of *Vachellia karroo* seedlings grown from seeds collected in three major river systems in the Southeastern Coastal Hinterland geomorphic province of South Africa. Error bars are standard errors of means.

**FIGURE 6:**
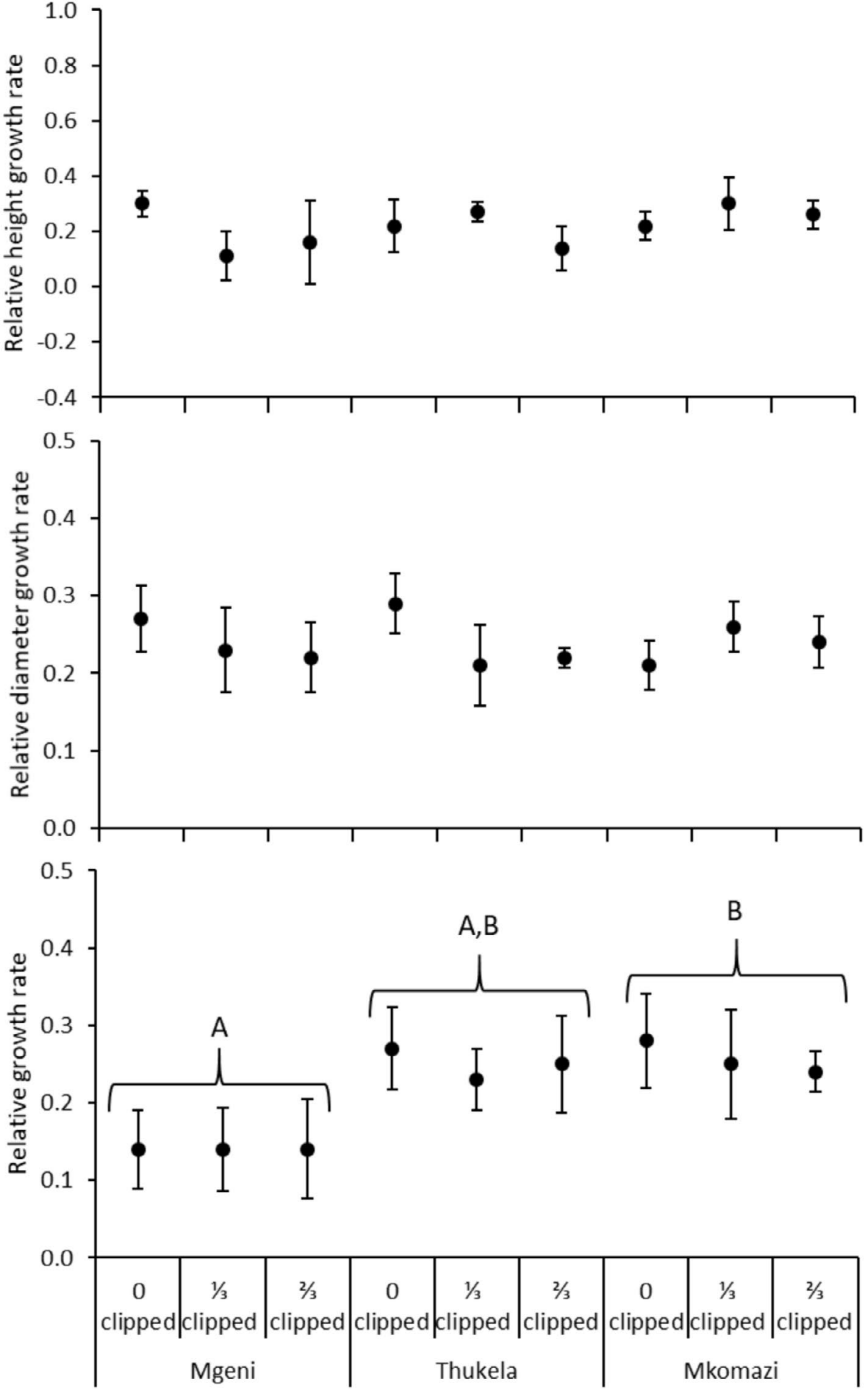
Mean relative height growth rate (mm mm^−1^ month^−1^), relative diameter growth rate (mm mm^−1^ month^−1^), and relative growth rate (mg mg^−1^ month^−1^) of *Vachellia karroo* seedlings grown from seeds collected in three major river systems in the Southeastern Coastal Hinterland geomorphic province of South Africa. Error bars are standard errors of means. Uppercase letters indicate significant differences between sites according to Tukey's HSD test (*p* < 0.05).

No significant interacting effects of clipping and site on response variables were detected in *V. karroo* seedlings (*F*_24,112.8_ = 0.660; *p* = 0.880; Wilk's Lambda = 0.633). Clipping had no significant effects on combined response variables (*F*_12,64_ = 0.546; *p* = 0.876; Wilk's Lambda = 0.823; Table 4; Figures 5 and 6), but site was significant (*F*_12,64_ = 2.149; *p* = 0.025; Wilk's Lambda = 0.508). Seedlings grown from seeds collected from Mkomazi grew faster than seedlings that originated from Mgeni (Table 4; Figure 6).

**TABLE 4:** Analysis of variance of leaf mass fraction (LMF), spine mass fraction (SMF), relative height growth rate (RHR, mm mm^−1^ month^−1^), relative diameter growth rate (RDR, mm mm^−1^ month^−1^), and relative growth rate (RGR, mg mg^−1^ month^−1^) of *V. karroo* seedlings (*n* = 47) grown in pots for 3 months following three clipping treatments applied at 3 months of age.

Dependent	Independent	Type III SS	df	Mean squares	*F*‐ratio	*p*
LMF	Clipping	0.012	2	0.006	0.467	0.631
	Site	0.051	2	0.025	2.010	0.148
	Clipping*Site	0.018	4	0.005	0.362	0.834
	Error	0.465	37	0.013		
SMF	Clipping	< 0.001	2	< 0.001	0.076	0.927
	Site	0.003	2	0.001	3.259	0.050
	Clipping*Site	0.001	4	< 0.001	0.742	0.570
	Error	0.015	37	0.001		
RHR	Clipping	0.013	2	0.006	0.187	0.830
	Site	0.061	2	0.031	0.909	0.412
	Clipping*Site	0.143	4	0.036	1.059	0.390
	Error	1.251	37	0.034		
RDR	Clipping	0.009	2	0.004	0.550	0.582
	Site	< 0.001	2	< 0.001	0.008	0.992
	Clipping*Site	0.031	4	0.008	0.974	0.433
	Error	0.296	37	0.008		
RGR	Clipping	0.001	2	< 0.001	0.020	0.981
	Site	0.119	2	0.059	3.812	0.031
	Clipping*Site	0.005	4	0.001	0.076	0.989
	Error	0.576	37	0.016		

## 4 Discussion

Support for our hypothesis, which predicted that spinescence increases and growth rate decreases after clipping, was limited to two species. While *V. nilotica* and *V. tortilis* seedlings could not compensate within 3 months for height lost by clipping because it reduced RHR, the absence of clipping effects on other growth metrics of both species suggested they could readily compensate for lost biomass. In comparison, *V. karroo* was able to compensate for both lost height and lost biomass. Given that all seedlings in this study, regardless of seed origin, were able to recover within 3 months of clipping, we hypothesized that compensation for lost spinescence was not at the expense of compensation for lost biomass in terms of carbon allocation (Scogings et al. 2013). This implies that seedlings of *V. nilotica* , *V. tortilis* , and *V. karroo*, regardless of seed origin in our study area, are well adapted to herbivory before the onset of the first dry season after germination, as long as herbivory is not later than the middle of the wet season in which they germinate.

The difference in responses of *V. nilotica* and *V. tortilis* versus *V. karroo* seedlings, whereby *V. karroo* seedlings compensated for lost height, but *V. nilotica* and *V. tortilis* seedlings did not, can be explained in terms of adaptations to herbivory or fire (Archibald and Bond 2003). Strong height regrowth would produce pole architecture and suggest adaptation of *V. karroo* to fire in association with herbivory or shading (Trollope 1984; Higgins et al. 2000; Allcock and Hik 2004; Vandenbussche et al. 2005; Stevens et al. 2007; Staver et al. 2009; Wakeling et al. 2011). Strong apical regrowth after herbivory results from activation of an axillary bud after browsing, which becomes the new leader shoot (Moncrieff et al. 2014). Weak vertical regrowth resulting from weak apical dominance results in cage architecture and suggests adaptation of *V. nilotica* and *V. tortilis* to herbivory (Vesey‐FitzGerald 1973; Bilbrough and Richards 1991; Archibald and Bond 2003; Charles‐Dominique et al. 2017). Increased branching is characteristic of cage architecture (Staver et al. 2012), but we did not record this. These differences in response to herbivory and fire have been well documented for saplings and adults (Archibald and Bond 2003; Staver et al. 2012), but have not been documented for seedlings in their first season of growth until now.

Responses to clipping were mostly independent of population, leading to the rejection of our prediction that populations of seedlings that grow slowly would increase spinescence and not compensate for lost height or biomass after clipping. The only site‐dependent response was increased spinescence in *V. tortilis* seedlings from Thukela, the slowest growing *V. tortilis* seedlings in the first 3 months after germination (Scogings and Mkhize 2023), but not over 6 months. After 6 months, seedlings of *V. nilotica* from Phongolo and Mfolozi were bigger than seedlings from Thukela, while seedlings of *V. karroo* from Mkomazi were bigger than seedlings from Mgeni. According to our hypothesis, seedlings of *V. nilotica* from Thukela and seedlings of *V. karroo* from Mgeni would be expected to overcompensate for lost spinescence and undercompensate for lost biomass after clipping, which did not happen. Our results for *V. nilotica* support the hypothesis that a strong evolutionary history of browsing has exerted strong selection for rapid growth in *V. nilotica* seedlings originating from Phongolo (Scogings and Mkhize 2023). However, explanations for our findings for *V. karroo* and *V. tortilis* are not yet clear. Moreover, seedlings of *V. karroo* from Thukela (where all species were collected) grew noticeably faster in 6 months than seedlings of *V. nilotica* and *V. tortilis* from Thukela, which supports the suggestion that *V. karroo* is well adapted to fire and herbivory, whereas *V. nilotica* and *V. tortilis* are well adapted to herbivory (discussed above).

While independent site effects were seen in *V. tortilis* , but not *V. karroo*, at 3 months (Scogings and Mkhize 2023), independent site effects were seen in *V. karroo*, but not *V. tortilis* , at 6 months. Among seedlings originating from Thukela, *V. nilotica* seedlings were the biggest at 3 months (Scogings and Mkhize 2023), but *V. karroo* seedlings were the biggest at 6 months. These observations suggest that seedlings of some species can switch between tolerance and defense while seedlings of other species maintain one or other strategy during the course of their inaugural growth season. Such differences among species may reflect differences in the timing of herbivory typically experienced by them in relation to their early ontogenetic trajectories. Such interspecific differences between seedlings of different ages could have implications for the assembly of woody plant communities. For example, a fire during the late wet season at a site where seedlings of all three species germinate in similar abundance could lead to the dominance of *V. karroo* over the other two species because of its stronger apical dominance.

In conclusion, we have demonstrated that certain traits adaptive to herbivory or fire in older life stages may be evident in the seedling stage, but that seedling age in certain species is important for detecting these traits. Second, we have shown that seedling age may be important to consider when determining differences in traits among different species' populations. We note that larger sample sizes would be beneficial for avoiding Type II errors in similar studies in the future. Overall, this study contributes to understanding plant responses to herbivory and fire and offers practical insights for managing these species in natural ecosystems.

## Author Contributions

**Peter F. Scogings:** conceptualization (equal), data curation (lead), formal analysis (lead), investigation (equal), methodology (equal), project administration (equal), visualization (lead), writing – original draft (lead), writing – review and editing (equal). **Ntuthuko R. Mkhize:** conceptualization (equal), formal analysis (supporting), funding acquisition (lead), investigation (equal), methodology (equal), project administration (equal), visualization (supporting), writing – original draft (supporting), writing – review and editing (equal).

## Conflicts of Interest

The authors declare no conflicts of interest.

## Data Availability

Data are available at the Zenodo repository (https://zenodo.org/records/13348850).

## References

[ece371163-bib-0001] Ackerly, D. D. , and M. J. Donoghue . 1998. “Leaf Size, Sapling Allometry, and Corner's Rules: Phylogeny and Correlated Evolution in Maples (Acer).” American Naturalist 152: 767–791. 10.1086/286208 18811427

[ece371163-bib-0002] Allcock, K. G. , and D. S. Hik . 2004. “Survival, Growth, and Escape From Herbivory Are Determined by Habitat and Herbivore Species for Three Australian Woodland Plants.” Oecologia 138: 231–241. 14576933 10.1007/s00442-003-1420-3

[ece371163-bib-0003] Archibald, S. , and W. J. Bond . 2003. “Growing Tall vs. Growing Wide: Tree Architecture and Allometry of *Acacia karroo* in Forest, Savanna, and Arid Environments.” Oikos 102: 3–14.

[ece371163-bib-0004] Archibald, S. , W. J. Bond , W. Hoffmann , C. Lehmann , C. Staver , and N. Stevens . 2020. “Distribution and Determinants of Savannas.” In Savanna Woody Plants and Large Herbivores, edited by P. F. Scogings and M. Sankaran , 3–24. John Wiley & Sons Ltd.

[ece371163-bib-0005] Archibald, S. , W. Twine , C. Mthabini , and N. Stevens . 2021. “Browsing Is a Strong Filter for Savanna Tree Seedlings in Their First Growing Season.” Journal of Ecology 109: 3685–3698.

[ece371163-bib-0006] Armani, M. , T. Charles‐Dominique , K. E. Barton , and K. W. Tomlinson . 2019. “Developmental Constraints and Resource Environment Shape Early Emergence and Investment in Spines in Saplings.” Annals of Botany 124: 1133–1142. 10.1093/aob/mcz152 PMC6943690 31560757

[ece371163-bib-0007] Augner, M. 1994. “Should a Plant Always Signal Its Defence Against Herbivores?” Oikos 70: 322–332.

[ece371163-bib-0008] Augustine, D. J. , P. F. Scogings , and M. Sankaran . 2020. “Mesobrowser Abundance and Effects on Woody Plants in Savannas.” In Savanna Woody Plants and Large Herbivores, edited by P. F. Scogings and M. Sankaran , 551–583. John Wiley & Sons Ltd.

[ece371163-bib-0009] Barton, K. E. 2008. “Phenotypic Plasticity in Seedling Defense Strategies: Compensatory Growth and Chemical Induction.” Oikos 117: 917–925.

[ece371163-bib-0010] Barton, K. E. 2016. “Low Tolerance to Simulated Herbivory in Hawaiian Seedlings Despite Induced Changes in Photosynthesis and Biomass Allocation.” Annals of Botany 117: 1053–1062. 27056973 10.1093/aob/mcw021 PMC4866310

[ece371163-bib-0011] Barton, K. E. , and K. Boege . 2017. “Future Directions in the Ontogeny of Plant Defence: Understanding the Evolutionary Causes and Consequences.” Ecology Letters 20: 403–411. 28145095 10.1111/ele.12744

[ece371163-bib-0012] Berenbaum, M. R. 1995. “Turnabout Is Fair Play: Secondary Roles for Primary Compounds.” Journal of Chemical Ecology 21: 925–940. 24234410 10.1007/BF02033799

[ece371163-bib-0013] Bilbrough, C. J. , and J. H. Richards . 1991. “Branch Architecture of Sagebrush and Bitterbrush: Use of a Branch Complex to Describe and Compare Patterns of Growth.” Canadian Journal of Botany 69: 1288–1295.

[ece371163-bib-0014] Boege, K. , R. Dirzo , D. Siemens , and P. Brown . 2007. “Ontogenetic Switches From Plant Resistance to Tolerance: Minimizing Costs With Age?” Ecology Letters 10, no. 3: 177–187. 17305801 10.1111/j.1461-0248.2006.01012.x

[ece371163-bib-0015] Briske, D. D. , and J. H. Richards . 1995. “Plant Responses to Defoliation: A Physiological, Morphological and Demographic Evaluation.” In Wildland Plants: Physiological Ecology and Developmental Morphology, edited by D. J. Bedunah and R. E. Sosebee , 635–710. Society for Range Management.

[ece371163-bib-0016] Brooks, R. , and N. Owen‐Smith . 1994. “Plant Defences Against Mammalian Herbivores: Are Juvenile *Acacia* More Heavily Defended Than Mature Trees?” Bothalia 24: 211–215.

[ece371163-bib-0017] Bryant, J. P. , F. S. Chapin , and D. R. Klein . 1983. “Carbon/Nutrient Balance of Boreal Plants in Relation to Vertebrate Herbivory.” Oikos 40: 357–368.

[ece371163-bib-0018] Charles‐Dominique, T. , J. Barczi , E. Le Roux , and S. Chamaillé‐Jammes . 2017. “The Architectural Design of Trees Protects Them Against Large Herbivores.” Functional Ecology 31: 1710–1717.

[ece371163-bib-0019] Chirara, C. 2001. “Tree Invasion in Semi‐Arid Savanna in Zimbabwe – Seedling Recruitment of *Acacia karroo*.” PhD thesis, Netherlands Utrecht University.

[ece371163-bib-0020] Comben, D. F. , G. A. McCulloch , K. Dhileepan , and G. H. Walter . 2021. “Genetic Identity of Australian Prickly Acacia (*Vachellia nilotica*, Fabales: Mimosoideae) – Assessing the Target for Biological Control.” Biological Control 155: 104540.

[ece371163-bib-0021] Ehrlich, P. R. , and P. H. Raven . 1964. “Butterflies and Plants: A Study in Coevolution.” Evolution 18: 586–608.

[ece371163-bib-0022] El Ferchichi Ouarda, H. , D. J. Walker , M. L. Khouja , and E. Correal . 2009. “Diversity Analysis of *Acacia tortilis* (Forsk.) Hayne Ssp. *Raddiana* (Savi) Brenan (Mimosaceae) Using Phenotypic Traits, Chromosome Counting and DNA Content Approaches.” Genetic Resources and Crop Evolution 56: 1001–1010.

[ece371163-bib-0023] Freeland, W. J. 1991. “Plant Secondary Metabolites: Biochemical Coevolution With Herbivores.” In Plant Defenses Against Mammalian Herbivory, edited by R. T. Palo and C. T. Robbins , 61–81. CRC Press.

[ece371163-bib-0024] Frost, P. G. H. , E. Medina , J. Menaut , O. Solbrig , M. Swift , and B. Walker . 1986. Responses of Savannas to Stress and Disturbance. International Union of Biological Sciences.

[ece371163-bib-0025] Goheen, J. R. , F. Keesing , B. F. Allan , D. Ogada , and R. S. Ostfeld . 2004. “Net Effects of Large Mammals on Acacia Seedling Survival in an African Savanna.” Ecology 85: 1555–1561.

[ece371163-bib-0026] Gols, R. , G. A. Desurmont , and J. A. Harvey . 2019. “Variation in Performance and Resistance to Parasitism of *Plutella xylostella* Populations.” Insects 10: 293. 31514415 10.3390/insects10090293 PMC6780392

[ece371163-bib-0027] Harborne, J. B. 1992. “The Chemical Basis of Plant Defense.” In Plant Defenses Against Mammalian Herbivory, edited by R. T. Palo and C. T. Robbins , 45–59. CRC Press.

[ece371163-bib-0028] Herms, D. A. , and W. J. Mattson . 1992. “The Dilemma of Plants: To Grow or Defend.” Quarterly Review of Biology 67: 283–335.

[ece371163-bib-0029] Heywood, J. S. 1991. “Spatial Analysis of Genetic Variation in Plant Populations.” Annual Review of Ecology and Systematics 22: 335–355.

[ece371163-bib-0030] Higgins, S. I. , W. J. Bond , and W. S. W. Trollope . 2000. “Fire, Resprouting and Variability: A Recipe for Grass‐Tree Coexistence in Savanna.” Journal of Ecology 88: 213–229.

[ece371163-bib-0031] Hoffmann, W. A. , and H. Poorter . 2002. “Avoiding Bias in Calculations of Relative Growth Rate.” Annals of Botany 90: 37–42. 12125771 10.1093/aob/mcf140 PMC4233846

[ece371163-bib-0032] Karban, R. , and I. T. Baldwin . 1997. Induced Responses to Herbivory. University of Chicago Press.

[ece371163-bib-0033] Kennedy, G. G. , and J. D. Barbour . 1992. “Resistance Variation in Natural and Managed Systems.” In Plant Resistance to Herbivores and Pathogens, edited by R. S. Fritz and E. L. Simms , 13–41. University of Chicago Press.

[ece371163-bib-0034] Knight, J. , and S. Grab . 2015. “The Drakensberg Escarpment: Mountain Processes at the Edge.” In Landscapes and Landforms of South Africa, edited by S. Grab and J. Knight , 47–55. Springer.

[ece371163-bib-0035] Koch, K. G. , K. Chapman , J. Louis , T. Heng‐Moss , and G. Sarath . 2016. “Plant Tolerance: A Unique Approach to Control Hemipteran Pests.” Frontiers in Plant Science 7: 1363. 27679643 10.3389/fpls.2016.01363 PMC5020058

[ece371163-bib-0036] Kyalangalilwa, B. , J. S. Boatwright , B. H. Daru , O. Maurin , and M. van der Bank . 2013. “Phylogenetic Position and Revised Classification of *Acacia* s.l. (Fabaceae: Mimosoideae) in Africa, Including New Combinations in *Vachellia* and *Senegalia* .” Botanical Journal of the Linnean Society 172: 500–523.

[ece371163-bib-0037] Lindroth, R. L. 1989. Plant‐Animal Interactions, edited by W. G. Abrahamson , 163–206. McGraw‐Hill.

[ece371163-bib-0038] Lindroth, R. L. , and S. B. St Clair . 2013. “Adaptations of Quaking Aspen (*Populus tremuloides* Michx.) for Defense Against Herbivores.” Forest Ecology and Management 299: 14–21.

[ece371163-bib-0039] Loveless, M. D. , and J. L. Hamrick . 1984. “Ecological Determinants of Genetic Structure in Plant Populations.” Annual Review of Ecology and Systematics 15: 65–95.

[ece371163-bib-0040] Massad, T. J. 2013. “Ontogenetic Differences of Herbivory on Woody and Herbaceous Plants: A Meta‐Analysis Demonstrating Unique Effects of Herbivory on the Young and the Old, the Slow and the Fast.” Oecologia 172: 1–10. 23053231 10.1007/s00442-012-2470-1

[ece371163-bib-0041] Mauricio, R. , M. D. Rausher , and D. S. Burdick . 1997. “Variation in the Defense Strategies of Plants: Are Resistance and Tolerance Mutually Exclusive?” Ecology 78: 1301–1311.

[ece371163-bib-0042] Milton, S. J. 1991. “Plant Spinescence in Arid Southern Africa: Does Moisture Mediate Selection by Mammals?” Oecologia 87: 279–287. 28313846 10.1007/BF00325267

[ece371163-bib-0043] Moles, A. T. , and M. Westoby . 2004. “Seedling Survival and Seed Size: A Synthesis of the Literature.” Journal of Ecology 92: 372–383.

[ece371163-bib-0044] Moncrieff, G. R. , S. Chamaillé‐Jammes , and W. J. Bond . 2014. “Modelling Direct and Indirect Impacts of Browser Consumption on Woody Plant Growth: Moving Beyond Biomass.” Oikos 123: 315–322.

[ece371163-bib-0045] Myers, J. H. , and D. Bazely . 1991. “Thorns, Spines, Prickles and Hairs: Are They Stimlated by Herbivory and Do They Deter Herbivores?” In Phytochemical Induction by Herbivores, edited by D. W. Tallamy and M. J. Raupp , 325–344. Wiley.

[ece371163-bib-0046] Omondi, S. F. , J. Machua , G. M. Muturi , J. M. Gicheru , and S. Hanaoka . 2019. “Evidence of High Genetic Diversity and Significant Population Structuring in *Vachellia tortilis* (Forsk.) Galasso & Banfi Population in Kenya.” Annals of Forest Science 76: 47–60.

[ece371163-bib-0047] Painter, R. H. 1951. Insect Resistance in Crop Plants. Macmillan.

[ece371163-bib-0048] Partridge, T. C. , E. S. J. Dollar , J. Moolman , and L. H. Dollar . 2010. “The Geomorphic Provinces of South Africa, Lesotho and Swaziland: A Physiographic Subdivision for Earth and Environmental Scientists.” Transactions of the Royal Society of South Africa 65: 1–47.

[ece371163-bib-0049] Partridge, T. C. , and R. R. Maud . 1987. “Geomorphic Evolution of Southern Africa Since the Mesozoic.” South African Journal of Geology 90: 179–208.

[ece371163-bib-0050] Pennings, S. C. 1996. “Testing for Synergisms Between Chemical and Mineral Defences – A Comment.” Ecology 77: 1948–1950.

[ece371163-bib-0051] Pollard, A. J. 1992. “The Importance of Deterrence: Responses of Grazing Animals to Plant Variation.” In Plant Resistance to Herbivores and Pathogens, edited by R. S. Fritz and E. L. Simms , 216–239. University of Chicago Press.

[ece371163-bib-0052] Rosenthal, J. P. , and P. M. Kotanen . 1994. “Terrestrial Plant Tolerance to Herbivory.” Trends in Ecology & Evolution 9: 145–148. 21236799 10.1016/0169-5347(94)90180-5

[ece371163-bib-0053] Rutherford, M. C. , L. Mucina , M. C. Lötter , et al. 2006. Savanna Biome. Vol. 19, 439–547. Strelitzia.

[ece371163-bib-0054] Rutherford, M. C. , L. Mucina , and L. W. Powrie . 2006. Biomes and Bioregions of Southern Africa. Vol. 19, 31–51. Strelitzia.

[ece371163-bib-0055] Scogings, P. F. , J. Hjältén , and C. Skarpe . 2013. “Does Large Herbivore Removal Affect Secondary Metabolites, Nutrients and Growth in Woody Species in Semi‐Arid Savannas?” Journal of Arid Environments 88: 4–8.

[ece371163-bib-0056] Scogings, P. F. , and N. R. Mkhize . 2023. “Seedling Growth and Spinescence of Three *Vachellia* Species' Populations in Valleys Formed by Tectonic Uplift.” African Journal of Ecology 61: 945–955.

[ece371163-bib-0057] Shaw, M. T. , F. Keesing , and R. S. Ostfeld . 2002. “Herbivory on *Acacia* Seedlings in an East African Savanna.” Oikos 98: 385–392.

[ece371163-bib-0058] Smith, T. M. , and S. E. Shackleton . 1988. “The Effects of Shading on the Establishment and Growth of *Acacia tortilis* Seedlings.” South African Journal of Botany 54: 375–379.

[ece371163-bib-0059] Staver, A. C. , W. J. Bond , M. D. Cramer , and J. L. Wakeling . 2012. “Top‐Down Determinants of Niche Structure and Adaptation Among African Acacias.” Ecology Letters 15: 673–679. 22507561 10.1111/j.1461-0248.2012.01784.x

[ece371163-bib-0060] Staver, A. C. , W. J. Bond , W. D. Stock , S. J. van Rensburg , and M. S. Waldram . 2009. “Browsing and Fire Interact to Suppress Tree Density in an African Savanna.” Ecological Applications 19: 1909–1919. 19831079 10.1890/08-1907.1

[ece371163-bib-0061] Stevens, M. T. , D. M. Waller , and R. L. Lindroth . 2007. “Resistance and Tolerance in *Populus tremuloides*: Genetic Variation, Costs, and Environmental Dependency.” Evolutionary Ecology 21: 829–847.

[ece371163-bib-0062] Stowe, K. A. , R. J. Marquis , C. G. Hochwender , and E. L. Simms . 2000. “The Evolutionary Ecology of Tolerance to Consumer Damage.” Annual Review of Ecology and Systematics 31: 565–595.

[ece371163-bib-0063] Strauss, S. Y. , and A. A. Agrawal . 1999. “The Ecology and Evolution of Plant Tolerance to Herbivory.” Trends in Ecology & Evolution 14: 179–185. 10322530 10.1016/s0169-5347(98)01576-6

[ece371163-bib-0064] Swemmer, A. , and D. Ward . 2020. “Patterns and Determinants of Woody Plant Growth in Savannas.” In Savanna Woody Plants and Large Herbivores, edited by P. F. Scogings and M. Sankaran , 331–438. John Wiley & Sons Ltd.

[ece371163-bib-0065] Taylor, C. L. , and N. P. Barker . 2012. “Species Limits in *Vachellia* (*Acacia*) *Karroo* (Mimosoideae: Leguminoseae): Evidence From Automated ISSR DNA “Fingerprinting”.” South African Journal of Botany 83: 36–43.

[ece371163-bib-0066] Tedder, M. , C. Morris , R. Fynn , and K. Kirkman . 2012. “Do Soil Nutrients Mediate Competition Between Grasses and *Acacia* Saplings?” Grassland Science 58: 238–245.

[ece371163-bib-0067] Tiffin, P. 2000. “Mechanisms of Tolerance to Herbivore Damage: What Do We Know?” Evolutionary Ecology 14: 523–536.

[ece371163-bib-0068] Tomlinson, K. W. , L. Poorter , F. Bongers , F. Borghetti , L. Jacobs , and F. van Langevelde . 2014. “Relative Growth Rate Variation of Evergreen and Deciduous Savanna Tree Species Is Driven by Different Traits.” Annals of Botany 114: 315–324. 24958787 10.1093/aob/mcu107 PMC4111386

[ece371163-bib-0069] Trollope, W. S. W. 1984. “Fire in Savanna.” In Ecological Effects of Fire in South African Ecosystems, edited by P. D. V. Booysen and N. M. Tainton , 199–218. Springer.

[ece371163-bib-0070] Trumble, J. T. , D. M. Kolodny‐Hirsch , and I. P. Ting . 1993. “Plant Compensation for Arthropod Herbivory.” Annual Review of Entomology 38: 93–119.

[ece371163-bib-0071] Tsvuura, Z. , and D. Ward . 2022. “Does a Reciprocal Transplant Experiment of Neighboring *Vachellia karroo* Populations Demonstrate Local Adaptation?” South African Journal of Botany 144: 316–324.

[ece371163-bib-0072] van der Meijden, E. , M. Wijn , and H. J. Verkaar . 1988. “Defence and Regrowth, Alternative Plant Strategies in the Struggle Against Herbivores.” Oikos 51: 355–363.

[ece371163-bib-0073] Vandenbussche, F. , R. Pierik , F. F. Millenaar , L. A. Voesenek , and D. Van Der Straeten . 2005. “Reaching out of the Shade.” Current Opinion in Plant Biology 8: 462–468. 16040269 10.1016/j.pbi.2005.07.007

[ece371163-bib-0074] Venter, Z. S. , M. D. Cramer , and H. Hawkins . 2018. “Drivers of Woody Plant Encroachment Over Africa.” Nature Communications 9: 2272. 10.1038/s41467-018-04616-8 PMC5995890 29891933

[ece371163-bib-0075] Vesey‐FitzGerald, D. F. 1973. “Animal Impact on Vegetation and Plant Succession in Lake Manyara National Park, Tanzania.” Oikos 24: 314–324.

[ece371163-bib-0076] Wakeling, J. L. , A. C. Staver , and W. J. Bond . 2011. “Simply the Best: The Transition of Savanna Saplings to Trees.” Oikos 120: 1448–1451.

[ece371163-bib-0077] Walker, B. H. 1985. “Structure and Function of Savannas: An Overview.” In Ecology and Management of the World's Savannas, edited by J. C. Tothill and J. J. Mott , 83–91. Australian Academy of Science and CAB.

[ece371163-bib-0078] Wang, I. J. , and G. S. Bradburd . 2014. “Isolation by Environment.” Molecular Ecology 23: 5649–5662. 25256562 10.1111/mec.12938

[ece371163-bib-0079] Wolde‐meskel, E. , and F. L. Sinclair . 1998. “Variations in Seedling Growth, Nodulation and Nitrogen Fixation of *Acacia nilotica* Inoculated With Eight Rhizobial Strains.” Forest Ecology and Management 104: 239–247.

[ece371163-bib-0080] Zangerl, A. R. , and F. A. Bazzaz . 1992. “Theory and Pattern in Plant Defense Allocation.” In Plant Resistance to Herbivores and Pathogens, edited by R. S. Fritz and E. L. Simms , 363–391. University of Chicago Press.

